# Supplementary material for: A comprehensive, improved protocol for generating common bean (Phaseolus vulgaris L.) transgenic hairy roots and their use in reverse-genetics studies
Source: PLoS One. 2024 Feb 21;19(2):e0294425. doi: 10.1371/journal.pone.0294425 (PMC10880956; doi:10.1371/journal.pone.0294425)
Supplement: S1 Table — (DOCX) [file pone.0294425.s001.docx]

|  | **Seed germination** | **Seedling growth** | **Growth of *A. rhizogenes*** | ***A. rhizogenes* infection** | **Hairy roots growth** | **Procedure for the selection of hairy roots** | **Use of the procedure** |
| --- | --- | --- | --- | --- | --- | --- | --- |
| **Original**  **protocol** | 2 days | 3 days | 3 days | In 5-days-old plants | ~15–days-old post infection | No included herein | Not discussed herein |
| **Updated protocol** | 2 days | Not required | ~30 h | In 2-days-old germinated seeds | ~12-days-old post infection | Included and discussed herein | Included and discussed herein |

**S 1 Table. Comparison of the original published protocol for the generation of transgenic hairy roots with the updated protocol described here, considering the critical steps.**
