# Supplementary material for: A comprehensive, improved protocol for generating common bean (Phaseolus vulgaris L.) transgenic hairy roots and their use in reverse-genetics studies
Source: PLoS One. 2024 Feb 21;19(2):e0294425. doi: 10.1371/journal.pone.0294425 (PMC10880956; doi:10.1371/journal.pone.0294425)
Supplement: S1 File — (PDF) [file pone.0294425.s002.pdf]

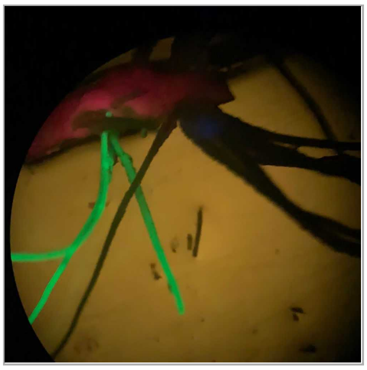

VERSION 2

JUN 07, 2023

OPEN ACCESS

DOI:
dx.doi.org/10.17504/protocols.io.261ge3bpjl47/v2

Protocol Citation: Ronal Pacheco, Georgina Estrada-Navarrete, Noreide Nava, Jorge Solis-Miranda, Carmen Quinto 2023. Hairy root generation in common bean (Phaseolus vulgaris L.) and selection of Agrobacterium rhizogenes clones. **protocols.io** https://dx.doi.org/10.17504/protocols.io.261ge3bpjl47/v2Version created by Ronal Pacheco

MANUSCRIPT CITATION:

License: This is an open access protocol distributed under the terms of the Creative Commons Attribution License, which permits unrestricted use, distribution, and reproduction in any medium, provided the original author and source are credited

Protocol status: Working
We use this protocol and it's working

Created: Jun 06, 2023

Hairy root generation in common bean (Phaseolus vulgaris L.) and selection of Agrobacterium rhizogenes clones V.2

Ronal Pacheco[1], Georgina Estrada-Navarrete[1], Noreide Nava[1], Jorge Solis-Miranda[1], Carmen Quinto[1]

[1]Instituto de Biotecnología, Universidad Nacional Autónoma de México

Carmen Quinto: Corresponding author

Ronal's protocols

Ronal Pacheco
Biotechnology Institute, National Autonomous University of M...

ABSTRACT

The common bean (*Phaseolus vulgaris* L.) is one of the legumes used to study the molecular mechanisms that regulate mycorrhizal and rhizobial symbioses. To study these mechanisms, the generation of transgenic hairy roots is a very advantageous method for applying genetic approaches. However, the generation of hairy roots is a difficult task that requires a lot of skill and experience. Here we show a new version of an optimized protocol for the generation of hairy roots in common bean. This version includes videos showing the procedure for preparing the *Agrobacterium rhizogenes* culture and the infection procedure, which were not included in the original version.

Last Modified: Jun 07, 2023

PROTOCOL integer ID:
82970

Seeds disinfection (when necessary)

1 Immerse the common bean seeds in [M] 96 % volume ethanol for 00:05:00 and wash them three times with sterile water. 5m

2 Immerse the seeds in [M] 2 % volume sodium hypochlorite for 00:05:00 and wash them three times with sterile water. 5m

Note

The concentration of sodium hypochlorite may be higher than indicated, but this depends on the quality of the seeds. When the quality of the seed is not very good, a higher concentration of sodium hypochlorite can damage a large number of them, rendering them useless for germination.

3 Keep the seeds at 4 °C in a disinfected container, e.g., sterile Plastic Petri dishes (100x15 mm) **Contributed by users**, or reused Petri dishes previously sterilized with [M] 96 % volume ethanol.

Seeds germination (1st day)

4 The disinfected seeds are placed, using sterile forceps, in a metal tray on a wet paper towel, previously sterilized in an autoclave, leaving 2 cm between the seeds.

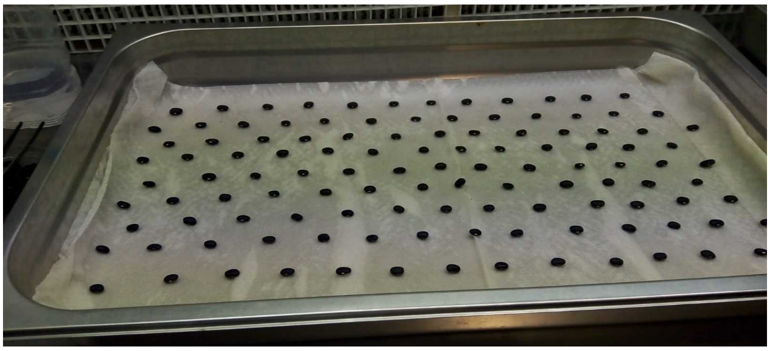

Note

Paper towels must be moistened with deionized and pre-autoclaved water.

5 Cover the metal tray with aluminum foil and incubate it in a growth chamber at 28 °C for 46:00:00 to 48:00:00 in the dark. 3d 22h

Note

Position the tray at a slight downward angle to improve seed germination. The hilum should face downward.

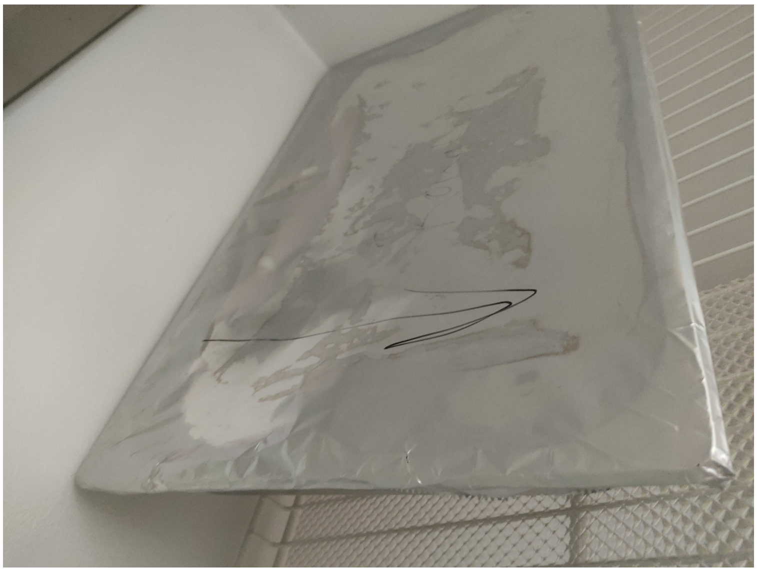

Preparation of the inoculum of A. rhizogenes K599 (2nd day)

6 Spread 150 µL - 200 µL of the inoculum in Petri dishes containing solid LB medium with the appropriate selection antibiotic.

Note

The inoculum consists of a liquid culture of *A. rhizogenes*, transformed with the corresponding vector, and 80% [M] 80 % volume glycerol [M] 50 % (v/v), stored at -80 °C. It is not recommendable to reuse the inoculum.

7 Incubate the Petri dishes inoculated in the previous step, for approximately 30:00:00 at 30 °C. 1d 6h

Note

If after 30 h the *A. rhizogenes* culture has not grown successfully, i.e., the culture layer is dry and very thin, do not use this inoculum for plant transformation and do the following:

7.1 Scratch off this thin layer of dried culture with a sterilized yellow tip or something similar.

Transfer this culture to an 1.5 mL Eppendorf tubes **Contributed by users** or 2 mL Eppendorf **Contributed by users**

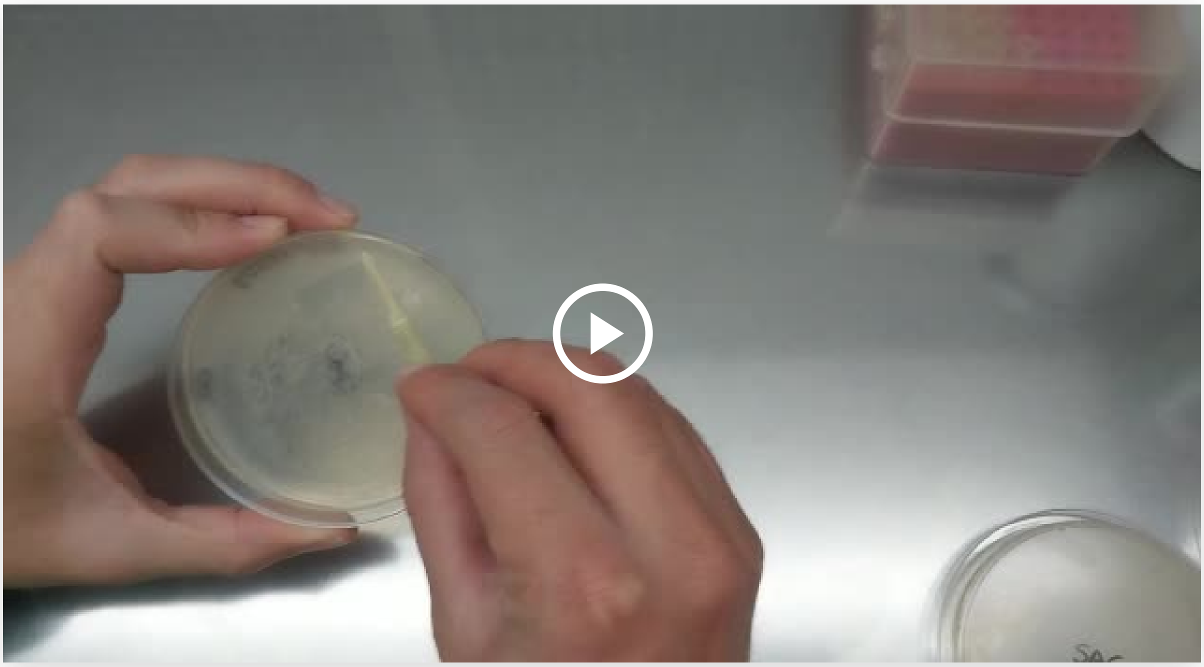

7.2 Add LB liquid medium to the Eppendorf tube and centrifugate 8000 rpm, Room temperature, 00:01:00 . Finally, homogenize the content using a micropipette. The content must be viscous, but liquid enough to be pipetted. 1m

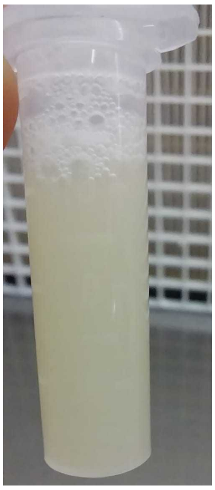

7.3 Prepare the inoculum in Eppendorf tubes (preferably 0.6 ml) by mixing an equal volume [M] 50 % (v/v) of the liquid content previously obtained, and [M] 80 % volume glycerol. Mix tubes by inversion and immediately place them in liquid nitrogen; finally, store the inoculum at -80 °C.

Note

To use this inoculum, spread 150 µL - 200 µL of the inoculum along with an equal volume of sterile LB liquid medium in Petri dishes containing solid LB medium with the appropriate selection antibiotic. Incubate for approximately 30:00:00 at 30 °C.

Seedling transformation by A. rhizogenes K599 (3rd day)

8 Carefully puncture the hypocotyl area of the seeds several times, using a sterile needle tip (0.4 mm). Apply the inoculum of *A. rhizogenes*The red on the wounded zone, taken directly from the plates, using an autoclaved micropipette tip.

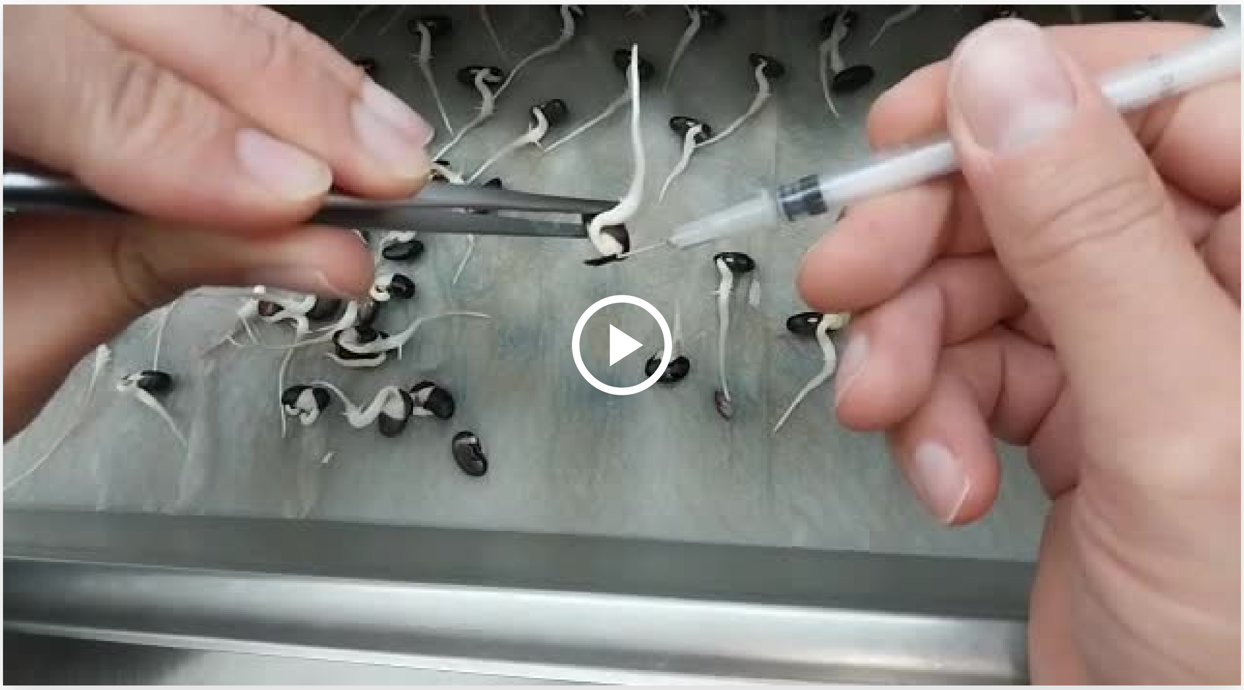

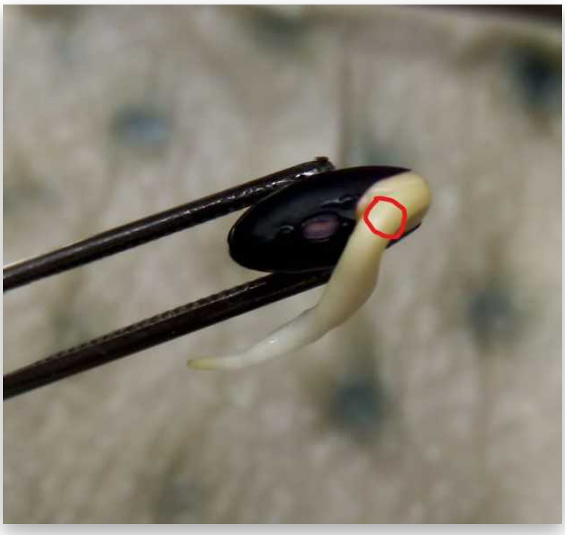

The red circle indicates the hypocotyl area.

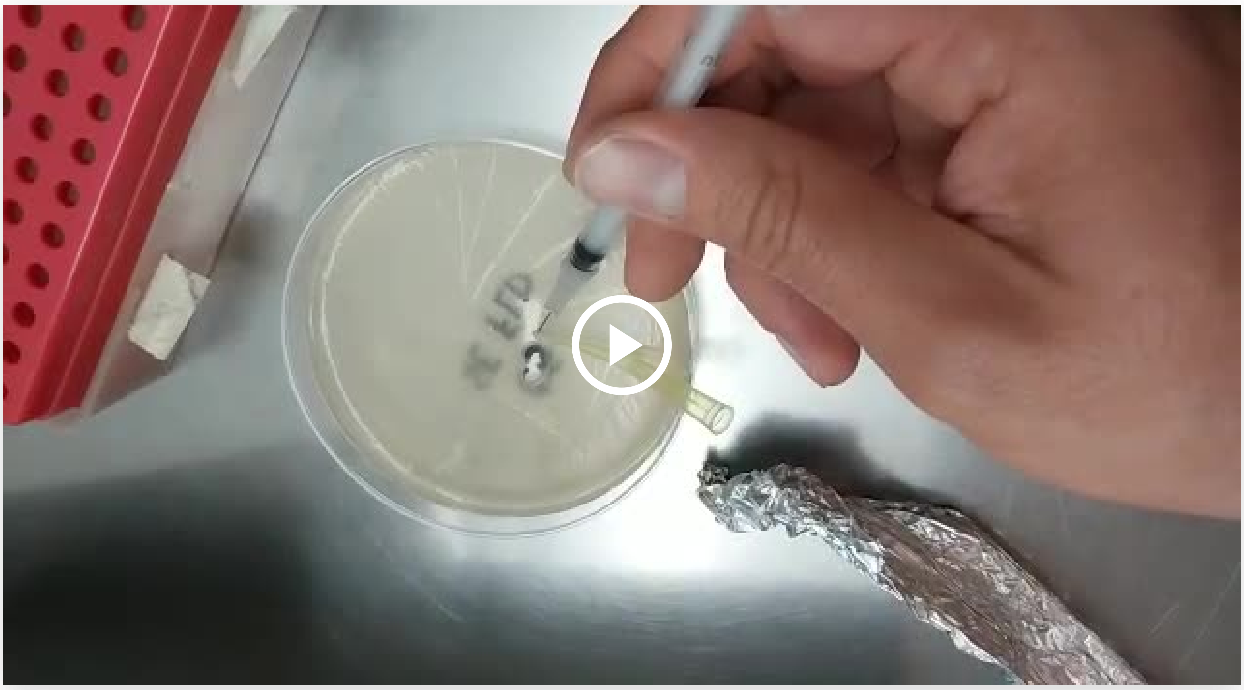

Note

Make sure that the hypocotyl tissue is not severely damaged. Damaged hypocotyl reduces seedling development and hairy root generation. The punctured area should not be too close or too far from the cotyledons.

Generation of hairy roots

9 Place the infected seedlings on the top of plastic tubes, i.e., 15 mL Falcon tubes **Contributed by users** containing B & D medium.

A. J. Márquez (Editorial Director). 2005. *Lotus japonicus* Handbook. pp. 53-82. https://link.springer.com/book/10.1007/1-4020-3735-X

Stock solutions	Solution component	2000x stock concentration	Concentration in final solution
A	$CaCl_2$	2.0 M	1.0 mM
B	KH_2PO_4	1.0 M	0.5 mM
C	Fe-citrate[b]	0.02 M	10 µM
D	$MgSO_4$	0.5 M	0.25 mM
E	K_2SO_4	0.5 M	0.25 mM
F	$MnSO_4$	2 mM	1.0 µM
G	H_3BO_3	4 mM	2.0 µM
H	$ZnSO_4$	1 mM	0.5 µM
I	$CuSO_4$	0.4 mM	0.2 µM
J	$CoSO_4$	0.2 mM	0.1 µM
K	Na_2MoO_4	0.2 mM	0.1 µM

Table 1. Broughton and Dilworth (1971) nutrient solution. [a]Adjust the pH to 6.8 with KOH. [b]Dissolve Fe-citrate with heating and keep in dark bottle

10 Place the plastic tubes inside glass tubes

PYREX Glass Rimless Test Tube 12x75mm **The Science Company® Catalog #NC-0993**

containing autoclaved deionized water and cover the glass tubes with plastic caps to prevent water evaporation.

11 Place glass tubes on racks and incubate in a growth chamber at 28 °C, 16:00:00 light/ 08:00:00 dark until hairy roots emerge, 10-12 days post-infection (dpi). 1d

Note

When the first pair of leaves reaches the plastic caps (3-5 dpi), remove the caps, and seal the tube hole with parafilm or adhesive plastic (image). During this period, make sure that the level of water and B & D medium contained within the glass tubes and plastics tubes, respectively, is adequate.

12 Once the hairy roots have emerged, remove the primary root by cutting the stem 1-2 cm below the hairy roots.

13 Transfer the seedlings to autoclaved glass tubes containing sterile B & D medium and seal the tube hole with parafilm or adhesive plastic.

Note

Make sure the level of the B & D medium is below the hairy root calluses, as covering hairy root calli with B & D medium may retard their growth.

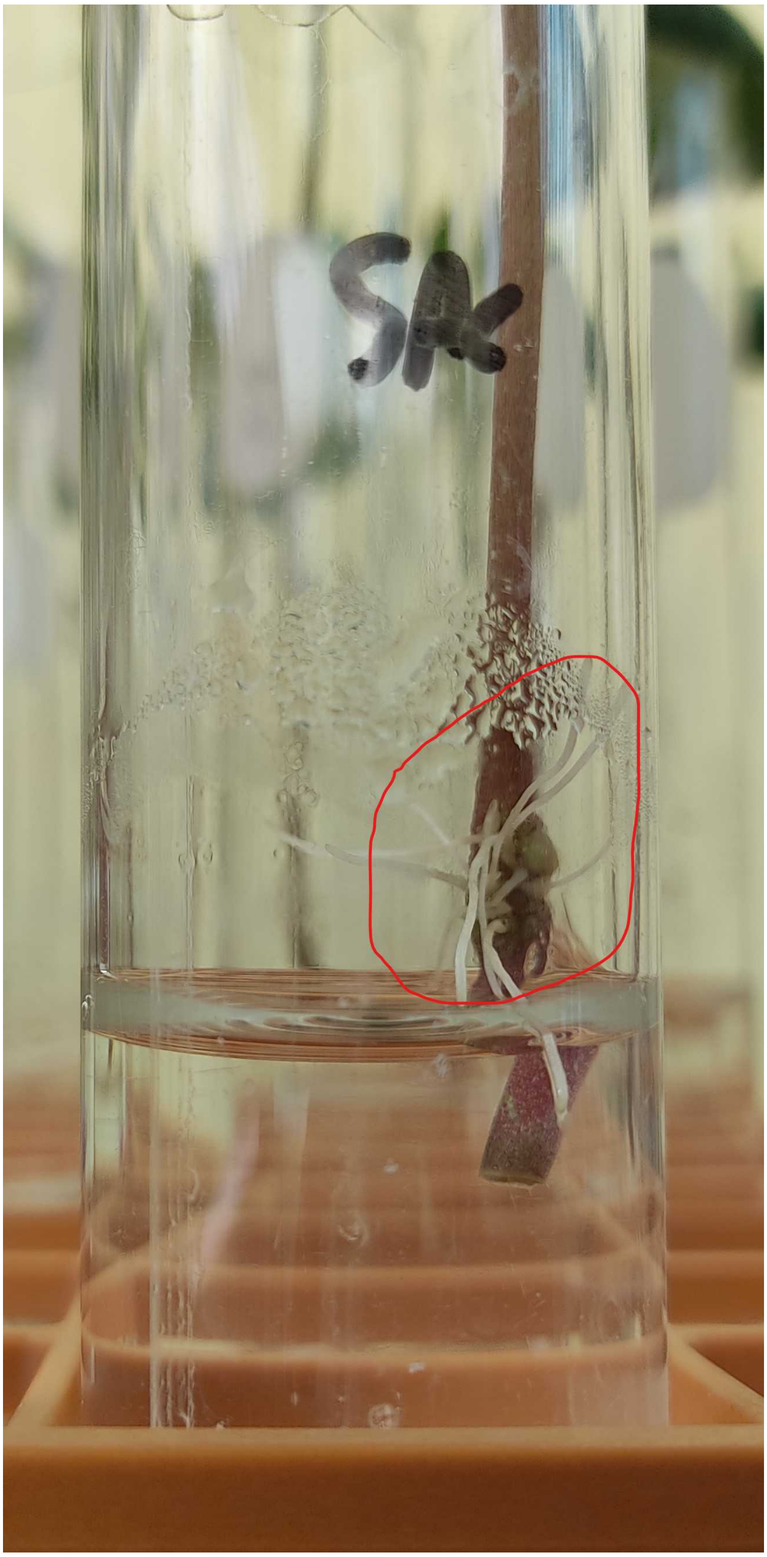

14 Incubate the seedlings for approximately three days under the same conditions described above to increase the biomass of hairy roots.

Selection of transformed hairy roots

15 Observe the fully developed hairy roots (15 to 16 dpi) using an epifluorescence microscope to remove non-fluorescent roots.

Note

Hairy roots must carry a plasmid containing a fluorescent reporter gene e.g., GFP, RFP, or YFP. Commonly, plasmids for RNAi-based gene silencing or gene overexpression carry a fluorescent reporter gene.

Selection of A. rhizogenes clones

16 Collect the fluorescent hairy roots carrying the RNAi-silencing or the overexpression vector, and the control vector, at the selected sampling time.

Note

If the amount of fluorescent hairy roots collected from a single plant is not enough tissue for RNA extraction, hairy roots from more than one plant will be needed.

17 Extract total RNA from hairy roots using an appropriate protocol and perform cDNA synthesis. We recommend using the following protocol dx.doi.org/10.17504/protocols.io.8epv5jq24l1b/v1

Protocol

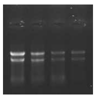

NAME

RNA extraction from hairy roots of common bean (Phaseolus vulgaris L.) and cDNA synthesis

CREATED BY

Ronal Pacheco

PREVIEW

18 Quantify transcript levels of the gene of interest by qPCR using two reference genes.

Note

We recommend as reference genes those encoding elongator factor 1α (*EF1α*, Phvul.004G075100.1) and β-tubulin (Phvul.009G017300.1). For RNAi silencing-based studies, we recommend choosing *A. rhizogenes* clones with a silencing efficiency of at least 70%.
