# Supplementary material for: A comprehensive, improved protocol for generating common bean (Phaseolus vulgaris L.) transgenic hairy roots and their use in reverse-genetics studies
Source: PLoS One. 2024 Feb 21;19(2):e0294425. doi: 10.1371/journal.pone.0294425 (PMC10880956; doi:10.1371/journal.pone.0294425)
Supplement: S2 File — (DOCX) [file pone.0294425.s003.docx]

**S2 Table. Values used to build graphs (Fig 1) by RAWGraphs (**[**https://app.rawgraphs.io/**](https://app.rawgraphs.io/)**).**

|  | **GUS-EGFP** | **OE-C3** | **pTdT-SAC** | **RNAi-C1** | **RNAi-C2** |
| --- | --- | --- | --- | --- | --- |
|  | -1,312 | -0,560 | 0,099 | 0,034 | 0,017 |
|  | -1,886 | -0,452 | 0,073 | 0,032 | 0,026 |
|  | -0,668 | 0,019 | 0,073 | 0,031 | 0,023 |
|  | -1,416 | 0,404 | 0,075 | 0,033 | 0,019 |
|  | -1,690 | 0,571 | 0,146 | 0,025 | 0,024 |
|  | -1,380 | 0,837 | 0,065 | 0,025 | 0,037 |
|  | -1,927 | 0,541 | 0,041 | 0,025 | 0,039 |
|  | -0,291 | 0,821 | 0,044 | 0,026 | 0,024 |
|  |  |  |  | 0,022 | 0,030 |
|  |  |  |  | 0,035 | 0,019 |
|  |  |  |  | 0,019 |  |
| **Q1** | -1.739 | -0.099 | 0.059 | 0.025 | 0.02 |
| **Q2** | -1.398 | 0.472 | 0.073 | 0.026 | 0.024 |
| **Q3** | -1.150 | 0.633 | 0.081 | 0.033 | 0.028 |
| **1.5IQR-Q1** | -1.92689 | -0.559 | 0.041 | 0.019 | 0.017 |
| **1.5IQR+Q3** | -0.29107 | 0.836 | 0.112 | 0.035 | 0.039 |

**S 3 Table. Results of statistical analysis of the abundance values of *pPLA-IIγ*
gene transcripts in transgenic roots, as shown in Fig 1.**

|  | **GUS-EGFP** | **OE-C3** | **pTdT-SAC** | **RNAi-C1** | **RNAi-C2** |
| --- | --- | --- | --- | --- | --- |
| **Median (Q2)** | -1.398 | 0.472 | 0.073 | 0.026 | 0.024 |
| ***Observed difference** |  | -1.87 |  | 0.047 | 0.049 |
| ****p-value** |  | 1e-04 |  | 3e-04 | 1e-04 |

*Observed differences between the median transcript abundance of pTdT-SAC and RNAi roots, before Montecarlo simulation.

**Probability of appearance of observed differences due to random sampling, after Montecarlo simulation.
