# Supplementary material for: A comprehensive, improved protocol for generating common bean (Phaseolus vulgaris L.) transgenic hairy roots and their use in reverse-genetics studies
Source: PLoS One. 2024 Feb 21;19(2):e0294425. doi: 10.1371/journal.pone.0294425 (PMC10880956; doi:10.1371/journal.pone.0294425)
Supplement: Attachment — Submitted filename: Response to Reviewers.docx [file pone.0294425.s005.docx]

**Reviewer Comments**

The article is a lab protocol in which, nutritionally and economically a very important crop, common bean (*Phaseolus vulgaris* L.) is under study for development of hairy roots. An already published data of one of the authors in 2007, showed significantly efficient hairy root cultivars as mentioned in the manuscript. However, the current study is a modified protocol of the original protocol with reverse genetics approaches included.

R. We really appreciate the reviewer's comments, which helped us improve our manuscript.

**Title**

The title of the manuscript should be more descriptive.

# R. We addressed your suggestion and modified the title as follows: “A comprehensive, improved protocol for generating common bean (*Phaseolus vulgaris* L.) transgenic hairy roots and their use in reverse-genetics studies”

**Abstract**

The sentence making and grammar errors must kept in consideration.

Proper punctuation is required in the abstract to make effective writing.

No results are described in the abstract.

R. We appreciate your feedback and have made the necessary changes to improve the wording in our manuscript. Since this report is not a classic article, but a protocol, it does not describe results as such, but "expected results." Therefore, a couple of sentences have been added that briefly describe the expected results.

**Introduction**

Please correct the grammatical errors

Introduction is very well written, however, the significance of reverse genetics approaches to understand the molecular mechanisms regulating mycorrhizal and rhizobial symbioses should be incorporated.

R. We appreciate this comment. The Introduction was modified based on this suggestion.

**Materials and Methods**

Not enough data about the plasmid construct and genes is given.

R. We agree with this comment. Information on plasmid construction has been added to the revised Materials and Methods section.

**Result**

The result section should be the outcomes of the experiments performed despite of the expected results.

In the results section, there is no description of relative abundance for gene expression explained (Fig 1).

There should be very explicit description of all the figures.

R. We appreciate the suggestions. We modified the text accordingly.

**Discussion**

Discussion section is very well explained, however, the literature insight on the results should be incorporated.

The discussion should have a strong link with the results explained

R. We agree with these comments and modified the text in the Discussion based on the suggestions.

The overall manuscript is comprised of a comprehensive approaches related to the already published lab protocols on common bean hairy root culture. This data on composite plants development and use of gene editing tools (CRISPR Cas9) could give better understanding about the gene function in common bean plants.

R. We greatly appreciate the comments, as they helped us improve our manuscript.
